# Supplementary material for: Thioredoxin is a metabolic rheostat controlling regulatory B cells
Source: Nat Immunol. 2024 Mar 29;25(5):873–85. doi: 10.1038/s41590-024-01798-w (PMC11065695; doi:10.1038/s41590-024-01798-w)
Supplement: Supplementary file 1 — Supplementary Figs. 1–4 and List 1. [file 41590_2024_1798_MOESM1_ESM.pdf]

nature immunology

Article

https://doi.org/10.1038/s41590-024-01798-w

Thioredoxin is a metabolic rheostat controlling regulatory B cells

In the format provided by the authors and unedited

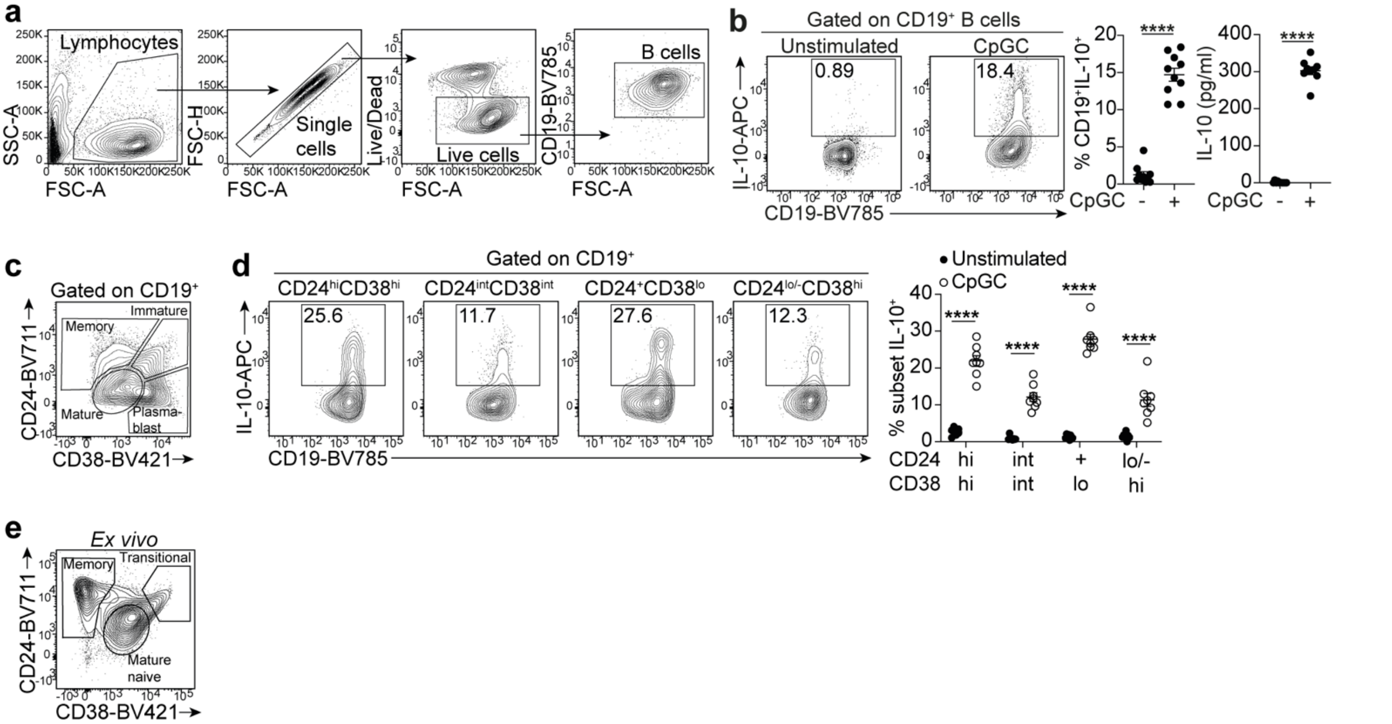

Supplementary Fig 1. Gating strategy for B cell identification and B cell subsets, and CpGC stimulation induces IL-10 production by B cells.

a, Gating strategy for identification of $CD19^{+}$B cells by flow cytometry. **b,** Representative contour plots and cumulative data show frequencies of $CD19^{+}IL\text{-}10^{+}$B cells and IL-10 secretion after 72h stimulation of isolated human B cells with CpGC. **c,** Gating strategy for $CD24^{hi}CD38^{hi}$ (immature), $CD24^{int}CD38^{int}$ (mature), $CD24^{+}CD38^{lo}$ (memory) B cells and $CD24^{lo/-}CD38^{hi}$ (plasmablasts) after 72h stimulation of isolated human B cells with CpGC. **d,** Representative contour plots and cumulative data show the frequencies of $IL\text{-}10^{+}$B cells within $CD24^{hi}CD38^{hi}$, $CD24^{int}CD38^{int}$, $CD24^{+}CD38^{lo}$ B cells and $CD24^{lo/-}CD38^{hi}$ plasmablasts after 72h CpGC stimulation. **e,** Gating strategy for fluorescence-activated cell sorting of transitional, mature naïve and memory B cells. ****P<0.0001 by two-tailed paired t-test (b) or two-way ANOVA with Sidak's correction for multiple comparisons (d). Error bars are shown as mean±SEM.

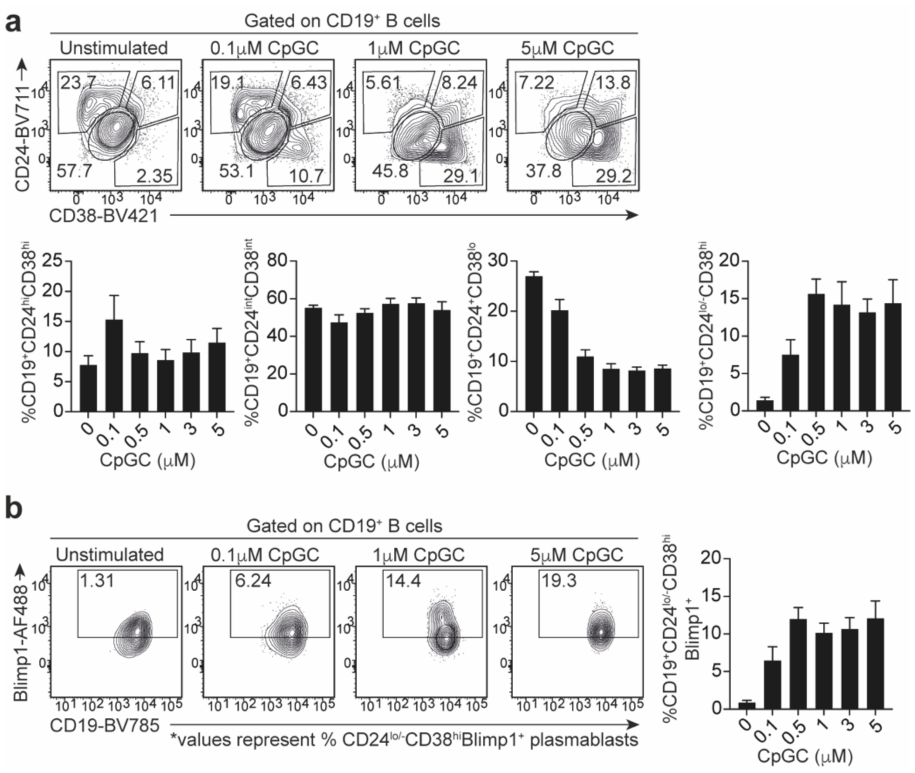

Supplementary Fig 2. In vitro stimulation of B cells with increasing concentrations of CpGC does not significantly alter the frequencies of B cell subsets.

a-b, representative contour plots and cumulative data show frequencies of (a) CD24^{hi}CD38^{hi} (immature), CD24^{int}CD38^{int} (mature), CD24^{+}CD38^{lo} (memory) B cells and CD24$^{lo/-}$CD38^{hi} (plasmablasts) and (b) CD24$^{lo/-}$CD38^{hi}Blimp1^{+} plasmablasts after 72h stimulation of isolated human B cells with increasing concentrations of CpGC.

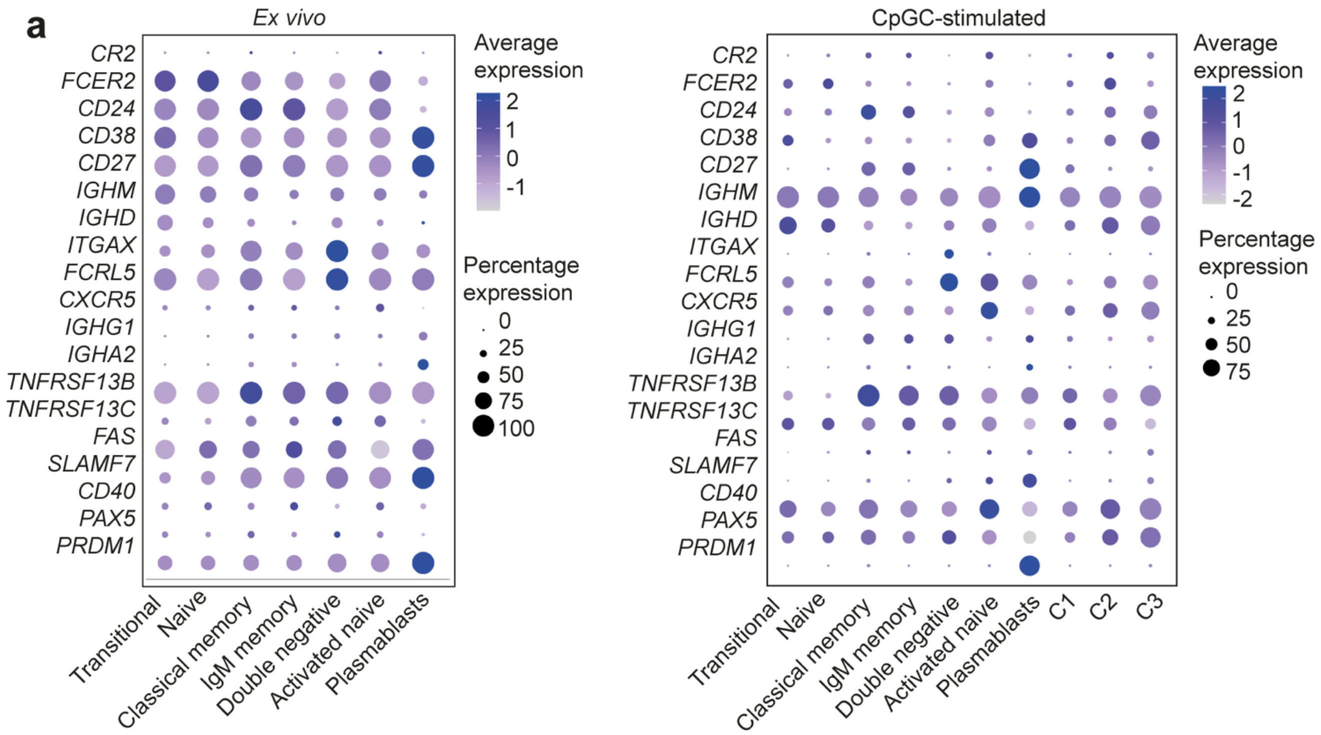

Supplementary Fig 3. Dotplots showing the expression of B cell phenotypic genes in (left) *ex vivo* B cells and (right) CpGC-stimulated B cells.

Supplementary List 1. Genes list reported in Breg cell signature (related to Fig. 3d)

DUSP2	*B3GNT2*	*SPAG9*	*SPCS3*	*RIF1*	*SLC38A5*	*ERLEC1*
IL10	*ARID5A*	*HSPA5*	*FAM107B*	*RRBP1*	*CEP290*	*GMNN*
MIR155HG	*LCP1*	*HM13*	*PDIA6*	*TUBB4B*	*SLC38A2*	*DNAJC1*
TNF	*NOP58*	*ARMH1*	*DNAJB11*	*CCDC28B*	*MACF1*	*GINS2*
CD83	*HSPE1*	*EHD4*	*ZNF667-AS1*	*KRAS*	*BORCS5*	*UBFD1*
NFKBID	*GNG2*	*SELENOS*	*PTP4A1*	*BRAF*	*YTHDC2*	*PDIA4*
IL21R	*RFTN1*	*DNAAF2*	*SREK1*	*SYAP1*	*MCTP2*	*CSKMT*
BCL2A1	*SLC39A8*	*SDF2L1*	*PPP1R16B*	*LGALS1*	*GMPR*	*NUP153*
SRGN	*LGALS3*	*INTS6*	*OGT*	*USP9X*	*CDT1*	*LSR*
SIAH2	*TXN*	*CYTOR*	*TNFRSF1B*	*ADA*	*SEC61A1*	*TIMP1*
DDX21	*LITAF*	*PTMS*	*ECE1*	*SLBP*	*IL6ST*	*FAM126A*
NFKB1	*LYST*	*RP9*	*GNG5*	*SKAP1*	*MICAL3*	*PPP1R10*
SEMA7A	*JPT1*	*HMCES*	*OSTC*	*MID1IP1*	*SPTBN1*	*PI4K2B*
CREM	*RPL22L1*	*ZNF267*	*IL15RA*	*P4HB*	*RPE*	*TK1*
CFLAR	*SEC61B*	*EIF4A3*	*SATB1*	*GABPB1*	*TSPYL2*	*HS2ST1*
LRMP	*DBI*	*CD1C*	*RANBP2*	*ELL2*	*JARID2*	*DNAJB1*
PTPN7	*COX5A*	*PRDX4*	*SLC4A7*	*UBR4*	*ATAD2B*	*TMEM99*
PPP1R15A	*SERTAD1*	*GPR137B*	*CCNH*	*SMC1A*	*AP1G1*	
LINC01480	*ARF4*	*PPP1R14B*	*HMGCS1*	*FKBP11*	*ACTB*	
FCRL5	*SLC7A5*	*MANF*	*HSPB1*	*ZNF318*	*LACTB*	
SERPINB9	*TUBA1B*	*CALR*	*TOR3A*	*DENND4A*	*MIDN*	
ABRACL	*SLAMF7*	*PIM1*	*IVNS1ABP*	*MACROD2*	*CEP95*	
IRF4	*HCST*	*TMED9*	*CDK6*	*NSF*	*BAZ2B*	
LDLRAD4	*S100A11*	*IFNGR1*	*YWHAH*	*SEC11C*	*CCNT2*	
GADD45B	*HSPH1*	*PFN1*	*CAMK2D*	*SMG7*	*BCOR*	
MT2A	*XBP1*	*ARPC1B*	*NEU1*	*BIRC2*	*GALNT2*	
SLC38A1	*ATG3*	*BIK*	*PNPT1*	*RUNX3*	*SEC14L1*	
ARPC5L	*MYDGF*	*C12orf75*	*ACTG1*	*ALG5*	*BRCA2*	
MAP3K8	*STMN1*	*HNRNPH1*	*RGS3*	*TUBG1*	*IFI30*	
NAB2	*FDX1*	*USP12*	*CRIP3*	*TYMS*	*CD2AP*	

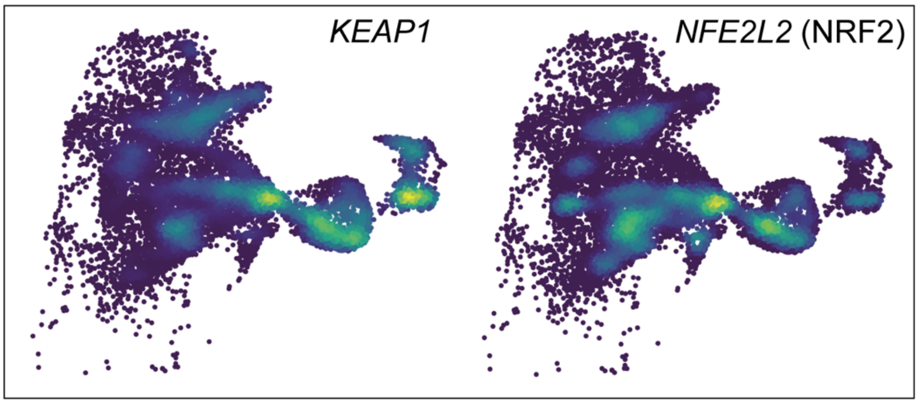

Supplementary Fig 4. scRNA-seq UMAPs show distribution of *KEAP1* and *NFE2L2* (encoding Nrf2)-expressing B cells.
