# Supplementary material for: Thioredoxin is a metabolic rheostat controlling regulatory B cells
Source: Nat Immunol. 2024 Mar 29;25(5):873–85. doi: 10.1038/s41590-024-01798-w (PMC11065695; doi:10.1038/s41590-024-01798-w)
Supplement: Supplementary file 2 — Reporting Summary [file 41590_2024_1798_MOESM2_ESM.pdf]

nature portfolio

Corresponding author(s): Claudia Mauri, Hannah Bradford

Last updated by author(s): 19/2/24

Reporting Summary

Nature Portfolio wishes to improve the reproducibility of the work that we publish. This form provides structure for consistency and transparency in reporting. For further information on Nature Portfolio policies, see our Editorial Policies and the Editorial Policy Checklist.

Statistics

For all statistical analyses, confirm that the following items are present in the figure legend, table legend, main text, or Methods section.

n/a	Confirmed	
☐	☒	The exact sample size (*n*) for each experimental group/condition, given as a discrete number and unit of measurement
☐	☒	A statement on whether measurements were taken from distinct samples or whether the same sample was measured repeatedly
☐	☒	The statistical test(s) used AND whether they are one- or two-sided<br>*Only common tests should be described solely by name; describe more complex techniques in the Methods section.*
☒	☐	A description of all covariates tested
☐	☒	A description of any assumptions or corrections, such as tests of normality and adjustment for multiple comparisons
☐	☒	A full description of the statistical parameters including central tendency (e.g. means) or other basic estimates (e.g. regression coefficient) AND variation (e.g. standard deviation) or associated estimates of uncertainty (e.g. confidence intervals)
☐	☒	For null hypothesis testing, the test statistic (e.g. *F*, *t*, *r*) with confidence intervals, effect sizes, degrees of freedom and *P* value noted<br>*Give P values as exact values whenever suitable.*
☒	☐	For Bayesian analysis, information on the choice of priors and Markov chain Monte Carlo settings
☒	☐	For hierarchical and complex designs, identification of the appropriate level for tests and full reporting of outcomes
☐	☒	Estimates of effect sizes (e.g. Cohen's *d*, Pearson's *r*), indicating how they were calculated

Our web collection on statistics for biologists contains articles on many of the points above.

Software and code

Policy information about availability of computer code

Data collection	FACS Diva v9
Data analysis	RStudio v4.2.1, Seurat v4.0, Monocle v3, Flowjo v10, Fiji v2.15.0

For manuscripts utilizing custom algorithms or software that are central to the research but not yet described in published literature, software must be made available to editors and reviewers. We strongly encourage code deposition in a community repository (e.g. GitHub). See the Nature Portfolio guidelines for submitting code & software for further information.

Data

Policy information about availability of data

All manuscripts must include a data availability statement. This statement should provide the following information, where applicable:

- Accession codes, unique identifiers, or web links for publicly available datasets
- A description of any restrictions on data availability
- For clinical datasets or third party data, please ensure that the statement adheres to our policy

All data are available in the main text or the supplementary materials. For scRNA-seq transcriptomic analysis, the GRCh38 reference genome was downloaded using the Bioconductor annotation package BSgenome.Hsapiens.NCBI.GRCh38. The scRNA-seq dataset has been deposited in ArrayExpress, under accession code E-MTAB-13872. Link: https://www.ebi.ac.uk/biostudies/arrayexpress/studies/E-MTAB-13872.

Research involving human participants, their data, or biological material

Policy information about studies with human participants or human data. See also policy information about sex, gender (identity/presentation), and sexual orientation and race, ethnicity and racism.

Reporting on sex and gender	We have used gender based on patient characteristics available to us.
Reporting on race, ethnicity, or other socially relevant groupings	Population (SLE and Healthy donor) characteristics are reported in Table 1
Population characteristics	Population (SLE and Healthy donor) characteristics are reported in Table 1<br><br>SLE participant and healthy donor (HD) demographics used in each experiment are as follows;<br>Age is reported as F:M<br><br>7a - (Sex) HD 5:8, SLE 1:27; (Avg Age) HD 41.5 SLE 46.8<br>7b - (Sex) HD 4:6, SLE 1:29 (Avg Age) HD 39.4, SLE 46.6<br>7c - (Sex) HD 2:8, SLE 1:17; (Avg Age) HD 37.1, SLE 42.9<br>7d - (Sex) HD 2:9, SLE 1:22; (Avg Age) HD 33, SLE 38.5<br>7e - (Sex) HD 3:8, SLE 1:27; (Avg Age) HD 31.2, SLE 46.8<br>7f - (Sex) HD 5:8, SLE 1:25; (Avg Age) HD 31.2, SLE 45.8<br>7g - (Sex) HD 4:6, SLE 1:24; (Avg Age) HD 29.4, SLE 46.9<br>7h - (Sex) HD 1:4. SLE 1:10; (Avg Age) HD 29, SLE 34.5<br>7i - (Sex) HD 3:8, SLE 1:15; (Avg Age) HD 39.4, SLE 39.1<br><br>Extended Data Fig 9a - (CELLROX) (Sex) HD 2:4, SLE 1:9; (Avg Age) HD 32.5, SLE 35.1 (MITOSOX) (Sex) HD 1:4, SLE 0:9; Avg Age) HD 30.2, SLE 34.3<br>Extended Data Fig. 9b - (Sex) HD 2:3, SLE 0:8; (Avg Age) HD 27.2, SLE 30.8<br>Extended Data Fig. 9c - (Sex) HD 2:3, SLE 0:7; (Avg Age) HD 33, SLE 33.8<br>Extended Data Fig. 9d - (Sex) HD 2:6, SLE 1:7; (Avg Age) HD 29.75, SLE 36.8<br>Extended Data Fig. 9e - (Sex) HD 2:6, SLE 1:8; (Avg Age) HD 29.75, SLE 35.1<br>Extended Data Fig. 9f - (Sex) HD 0:3, SLE 0:3; (Avg Age) HD 30, SLE 36.6
Recruitment	SLE patients and healthy donors were recruited following informed consent. SLE patients were recruited from the University College London Hospital (UCLH) Rheumatology outpatient clinic. All patients recruited met at least 4 of the 11 American College of Rheumatology classification criteria, with a disease duration lasting more than 6 months, and positivity for antinuclear antibody (ANA) or anti-double-stranded DNA (dsDNA) autoantibodies. Patients under the age of 18, those treated with B cell depleting therapies (including rituximab, belimumab), those participating in any interventional trial, or pregnancy, were excluded from the study. Also excluded were patients with severe CNS lupus, glomerulonephritis, or congestive heart failure, and patients with a history of infections including HIV, hepatitis B/C and tuberculosis. Patients alto meeting criteria for other autoimmune diseases (such as multiple sclerosis, rheumatoid arthritis) were excluded. Participants did not receive compensation. Participants were recruited based on these criteria and attendance to the UCLH Rheumatology outpatients clinic. Healthy donors included research team co-workers. No self-selection bias that could impact results has been identified.
Ethics oversight	UCLH Health Service Trust ethics committee - REC reference no. 14/SC/1200

Note that full information on the approval of the study protocol must also be provided in the manuscript.

Field-specific reporting

Please select the one below that is the best fit for your research. If you are not sure, read the appropriate sections before making your selection.

☒ Life sciences ☐ Behavioural & social sciences ☐ Ecological, evolutionary & environmental sciences

For a reference copy of the document with all sections, see nature.com/documents/nr-reporting-summary-flat.pdf

Life sciences study design

All studies must disclose on these points even when the disclosure is negative.

Sample size	No statistical methods were used to predetermine sample sizes, but our sample sizes are similar to those reported in previous publications (Shankar 2022; https://doi.org/10.1038/s41467-022-30613-z, Menon 2016; https://doi.org/10.1016/j.immuni.2016.02.012, Lighaam 2018; https://doi.org/10.3389/fimmu.2018.01913)
Data exclusions	All our data points have been included.
Replication	Experiments were repeated twice if sample size was >=6 or three times if sample size was between 3 and 6. All attempts at replication were successful

Randomization	No randomisation criteria could be applied. People were chosen according to disease (SLE or healthy donors), and allocated into these two groups.
Blinding	Data collection and analysis were not performed blind to the conditions of the experiments as all biological samples were labeled.

Reporting for specific materials, systems and methods

We require information from authors about some types of materials, experimental systems and methods used in many studies. Here, indicate whether each material, system or method listed is relevant to your study. If you are not sure if a list item applies to your research, read the appropriate section before selecting a response.

Materials & experimental systems

n/a	Involved in the study	
☐	☒	Antibodies
☒	☐	Eukaryotic cell lines
☒	☐	Palaeontology and archaeology
☒	☐	Animals and other organisms
☒	☐	Clinical data
☒	☐	Dual use research of concern
☒	☐	Plants

Methods

n/a	Involved in the study	
☒	☐	ChIP-seq
☐	☒	Flow cytometry
☒	☐	MRI-based neuroimaging

Antibodies

Antibodies used

BV785-conjugated mouse anti-human CD19 (Biolegend, Cat# 302239, clone HIB19, lot# B373234).
BV711-conjugated mouse anti-human CD24 (Biolegend, Cat# 311135, clone ML5, lot# B376031)
BV421-conjugated mouse anti-human CD38 (Biolegend, Cat# 356617, clone HB7, lot# B346969)
APC-conjugated rat anti-human IL-10 (Biolegend, Cat# 506807, clone JES3-19F1, lot# B370178)
PE-conjugated rat anti-human IL-6 (Biolegend, Cat# 501106, clone MQ2-13A5, lot# B365304)
PE/Cy7-conjugated mouse anti-human LAP(TGFb) (Biolegend, Cat# 300007, clone S20006A, lot# B371003)
PE-conjugated mouse anti-human IL-27/IL-35 (Biolegend, cat# 360903, clone B032F6, lot# B371876)
PE/Dazzle-conjugated rat anti-human GM-CSF (Biolegend, cat# 502317, clone BVD2-21C11, lot# B377133)
FITC-conjugated mouse anti-human IL-12/IL-35 p35 (R&D Systems, cat# IC2191F clone 27537 lot# LIR0721051)
AlexaFluor488-conjugated mouse anti-human Blimp1/PRDM1 (R&D Systems, cat# IC36081G, clone 646702, lot# ACTS0420031)
eFluor450-conjugated mouse anti-human TNF (ThermoFisher Scientific, cat# 48-7349-42, clone Mab11, lot# B370178)
eFluor450-conjugated mouse anti-human IFNg (ThermoFisher Scientific, cat# 48-7139-42, clone 4S.B3, lot# 273837
BUV395-conjugated mouse anti-human Ki67 (BD, cat# 564071, clone B56, lot# 1102683)
BUV395-conjugated mouse anti-human CD3 (BD, cat# 563546, clone UCHT-1, lot# 1214287)
PE-conjugated mouse anti-human CD4 (Biolegend, cat# 344605, clone SK3, lot# B303125)
Mouse anti-human thioredoxin (BioRad, cat# VMA00585, clone 3A1, lot# 161202)
Rabbit anti-human thioredoxin-2 (Abcam, cat# ab18554, clone EPR15225, lot# GR325111)

All surface marker antibodies (CD19, CD24, CD38, CD3, CD4) were used at 1:50 dilution.
All intracellular and intranuclear antibodies (IL-10, IL-6, TNF, IFNg, TGFb, IL-27/IL-35, IL-12/IL-35 p35, GM-CSF, Ki67, Blimp1, Trx, Trx2) were used at 1:100 dilution.

Validation

All antibodies have been validated by other publications and/or the manufacturer.

Plants

Seed stocks	NA
Novel plant genotypes	NA
Authentication	NA

Flow Cytometry

Plots

Confirm that:

☒ The axis labels state the marker and fluorochrome used (e.g. CD4-FITC).

☒ The axis scales are clearly visible. Include numbers along axes only for bottom left plot of group (a 'group' is an analysis of identical markers).

☒ All plots are contour plots with outliers or pseudocolor plots.

☒ A numerical value for number of cells or percentage (with statistics) is provided.

Methodology

Sample preparation	B cells were isolated from PBMCs and LNs using the EasySep™ immunomagnetic negative selection kit (STEMCELL, 19054). Isolated B cells were cultured for 72h with CpGC ODN 2395 (1µM, Invivogen tlrl-2395-1) in RPMI 1640 containing L-glutamine (Sigma-Aldrich). Media were supplemented with 10% fetal calf serum (FCS; LabTech) and 1% penicillin/streptomycin (100U/ml penicillin + 100µg/ml streptomycin; Sigma-Aldrich). To facilitate intracellular cytokine staining, cells were cultured in complete medium with PMA (50ng/ml; Sigma-Aldrich, P1585), ionomycin (250ng/ml, Sigma-Aldrich, I9657) and Brefeldin (5µg/ml; Sigma-Aldrich, B7651) for 4.5h. For multi-colour flow cytometric cell surface staining, cells were stained at 4oC for 30 minutes. LIVE/DEAD fixable blue Dead Cell Stain (ThermoFisher, L23105) was used to exclude dead cells from analysis. Cells were intracellularly fixed and permeabilised (ThermoFisher Scientific, 88-8824-00), or for additional detection of Blimp1 and Ki67 cells were fixed for 30 minutes using the FoxP3 Fixation buffer kit (ThermoFisher Scientific, 00-5523-00) before permeabilization. Cells were then incubated with intracellular and intranuclear antibodies for 30 minutes at 4oC.
Instrument	Flow cytometer: BD Pharmingen LSR II<br>Sorter: BD FACSAria Fusion
Software	BD FACS Diva v9.0 was used for data acquisition, Flowjo v10 was used for flow cytometry data analysis.
Cell population abundance	Reported values in the figures are percentages of either total CD19+B cells, or percentages of B cell phenotypic subsets
Gating strategy	Acquired samples were gated on FSC-A/SSC-A to select lymphocytes, FSC-H/FSC-A to exclude cell doublets, then cells negatively staining for Live/Dead Blue to exclude dead cells. Cells were then gated as FCS-A/CD19-BV785+ to identify B cells. All subsequent gating (e.g. CD24/CD38 to identify B cell subsets, IL-10+ B cells) were performed within the CD19+ gate, or for some figures IL-10+ or Blimp1+ cells were gated within CD24/CD38 B cell subsets.

☒ Tick this box to confirm that a figure exemplifying the gating strategy is provided in the Supplementary Information.
